# The association between physical activity and quality of life among people aged 60–89 living in own homes and nursing homes

**DOI:** 10.1186/s12877-024-04898-2

**Published:** 2024-03-23

**Authors:** Aleksandra Kiełtyka-Słowik, Urszula Michalik-Marcinkowska, Bożena Zawadzka

**Affiliations:** 1 Department of Health Promotion, Institute of Basic Sciences, University of Physical Education in Cracow, Cracow, Poland; 2 Department of Family Medicine and Public Health, Faculty of Medicine, University of Opole, Opole, Poland; 3 Faculty of Pedagogy and Psychology, Institute of Pedagogy, Jan Kochanowski University of Kielce, Kielce, Poland

**Keywords:** Physical activity, Quality of life, Older people, IPAQ, WHOQOL-AGE, Nursing home

## Abstract

**Background:**

The aim of the study was to obtain a response to the question of whether and how physical activity (PA) among people aged 60–89 years impacts quality of life and other sociodemographic characteristics (sex, age and place of living).

**Methods:**

Among 341 respondents aged 60 to 89, including 273 women (80%) and 68 men (20%) successfully completed IPAQ and WHOQOL AGE questionnaires. In the study were used International Physical Activity Questionnaire – IPAQ and World Health Organization Quality Of Life - Age – WHOQOL-AGE in Polish version.

**Results:**

The average total physical activity, including vigorous-intensity physical effort, moderate-intensity physical effort and walking amounts to 1381.87 ± 1978.60 MET-min/week. The average quality of life for the whole group of older people as evaluated with WHOQOL AGE scale was 64.79 (SD = 14.76; min:18.77-max: 98.07). Statistical analysis between physical activity and life quality proved significant dependence for the global life quality rating (*p* < 0.001).

**Conclusions:**

Our research has shown that PA improves quality of life among older people. Higher scores of quality of life were obtained in the F1 subscale (satisfaction) than in the F2 subscale (meeting expectations) in both age groups. Age significantly affects quality of life for older people.

## Introduction

The latest guidelines published by the American Heart Association (AHA) and American College of Cardiology (ACC) indicate the importance of physical activity in maintaining and improving health [1]. Physical activity provides multiple health, economic and social benefits, while active recreation (including walking, cycling or doing housework) may contribute directly to achieving sustainable development goals as defined by the World Health Organization in the Global Action Plan on Physical Activity 2018–2030 [2], which is essential not only for the current population but also for future generations. Hence, physical activity is significant both for an individual and the whole population and society in the context of a generational viewpoint.

Regular physical activity can reduce the risk of numerous diseases in older adults,boosts efficiency and physical capacity, aids successful aging, enhances healthiness, promotes proper eating habits and improves quality of life [3–6]. Moreover, moderate-intensity physical activity for 3 h weekly can decrease the risk of death by 27% [7]. Carlson et al., on the other hand, attributed the risk of premature death as high as 8.3% to the lack of physical activity [8]. Feng et al. indicated that the recommendations concerning 150 min of both moderate-intensity and vigorous-intensity physical activity weekly statistically improved quality of life (QOL), enhanced cognitive functions and reduced symptoms of depression in older adults aged 60 and over [9]. The idea of quality of life is a broad concept tightly connected with life satisfaction. Additionally, quality of life is frequently analysed with reference to the aging process. The literature proves that everyday activity, family support, good sleeping quality, no drinking alcohol and having a good sense of sight and hearing altogether may determine QOL [10].

The aim of the study was to obtain a response to the question of whether and how physical activity among people aged 60–89 years impacts quality of life and other sociodemographic characteristics (sex, age and place of living). It was conducted the first cross-sectional and comparable study in Poland using two questionnaires that are dedicated to older people: IPAQ (International Physical Activity Questionnaire) short version and the WHOQOL-AGE (World Health Organization Quality of Life - Age).

## Methods

### The characteristics of participants

A group of 600 people aged 60–89 was surveyed. Of the 600 people surveyed, only 341 met the inclusion criteria for the study (the inclusion criteria are described below in the Procedure section). As a result, were taken into account for further analyses 341 respondents, including 273 women (80%) and 68 men (20%), successfully completed the IPAQ and WHOQOL AGE questionnaires. Among the researched group, 219 (64%) people were aged 60–74 years, and 122 (36%) were aged 75–89. Moreover, 178 (52%) were living in their own homes, and 163 (48%) were living in an institution (nursing homes).

### Tools

The quality of life of the questioned people was assessed by means of the WHOQOL-AGE questionnaire adapted to the Polish version (Cronbach’s alpha coefficient was 0.89 for the first factor and 0.85 for the second). This questionnaire covers 13 questions, which are designed to self-evaluate health and well-being, feelings of loneliness and degree of social cohesion. The initial WHOQOL-AGE scale was based on both classical test theory (CTT) and item response theory (IRT). In this way, the hierarchic factor structure of the scale was created and consisted of one second-rate factor and two first-rate factors. The first factor includes questions from Q1 to Q8 (subscale F1 – assessing the level of satisfaction), and the following factor includes questions from Q9 to Q13 and Q1 (subscale F2 – assessing meeting expectations). The final result is an arithmetic average calculation of the F1 and F2 subscales. The WHOQOL-AGE questionnaire determines the level of QOL on a 0-100 scale [11, 12]. The authors of the abovementioned questionnaire expressed their consent for the application of the tool to the study.

The intensity of physical activity performed by the older people was assessed by means of the International Physical Activity Questionnaire IPAQ. A short version of this questionnaire expresses physical activity in MET min per week units (MET, metabolic equivalent) and allows us to classify the respondents on the basis of the physical activity they undertake into three categories: insufficient level of physical activity (below 600 MET min/week), satisfactory level of physical activity (600–1500 or 600 − 300 MET min/week) and high level of physical activity (above 1500 or 3000 MET min/week). This questionnaire consists of six short closed questions and seven subsequent questions regarding vigorous-intensity physical effort, moderate-intensity physical effort, walking and time spent sitting. Walking, vigorous-intensity physical effort and moderate-intensity physical effort comprise the so-called total physical activity, whereas time spent sitting is defined as a separate physical activity class [13]. The authors of the abovementioned questionnaire expressed their consent for the application of the tool to the study.

### Procedure

The selection of the test group was single-stage and intentional. The heads of the nursing homes in Cracow and the Universities of Third Age in Cracow had been informed about the aim and scope of the research and expressed their consent to conduct the study. Also every participant was informed about the aim of study. Only the respondents who had provided written informed consent beforehand were questioned. The questionnaires were distributed among older people, and they individually filled them in. Respondents were given questionnaires in paper form.

The following inclusion criteria were adopted: people aged 60–89 residing in nursing homes or attending the courses of the University of Third Age, physically capable, and scoring above 10 points on the IADL (Instrumental Activities of Daily Living Scale) scale. The following exclusion criteria were adopted: lack of written consent of the respondents or a head of nursing home, missing personal details, limited communication capacity or no communication capacity due to mental health problems (scoring less than 24 points on Mini-Mental State Examination), incomplete completion of the questionnaire.

For participants from nursing homes, the Mini-Mental State Examination scale was included in the medical records, which were analyzed by the authors of the study in terms of sample selection, while the Instrumental Activities of Daily Living Scale was included in the main questionnaire. For U3A participants, the Mini-Mental State Examination and Instrumental Activities of Daily Living Scale were included in the general part of the questionnaire.

### Statistical analysis

For statistical analysis, the Statistica version 13.3 (TIBCO Software Inc, 2017) program was used. First, all results were entered manually into the spreadsheet and accumulated in Microsoft Excel. Next, descriptive statistics were calculated: counts, arithmetic mean, standard deviation and median. After checking normality by the Shapiro‒Wilk test, nonparametric Mann–Whitney U (for two variables) or Kruskal‒Wallis (for three variables) tests were used to identify the significance of intergroup differences in the values of the analysed measurable variables. To correlate the variables on an ordinal scale, the Spearman rank correlation coefficient was used. The statistical significance *p* < 0.05 was adopted.

### Ethics

The Bioethics Committee in Regional Medical Chamber in Kraków officially issued a permission to conduct research no 88/KBL/OIL/2018 on 8th May 2018. Informed consent was obtained from all participants.

## Results

### Level of physical activity

The average total physical activity, including vigorous-intensity physical effort, moderate-intensity physical effort and walking was 1381.87 ± 1978.60 MET-min/week. Walking constitutes the greatest share of total physical activity for older people (731.11 ± 1054.29 MET-min/week), followed by moderate-intensity physical effort (406.19 ± 902.72 MET-min/week) and vigorous-intensity physical effort (300.05 ± 1015.25 MET- min/week). The respondents spent the least time sitting (208.39 ± 118.45 MET-min./week).

Among the studied people, 19% (66 people) of respondents reported performing vigorous-intensity physical activity, whereas 60% of the surveyed (206 people) reported undertaking moderate-intensity physical effort, the remaining 21% (69 people) had no physical activity.

Almost half of the respondents, 164 (48%), were classified and marked as insufficiently physically active according to the IPAQ questionnaire, 120 people (35%) scored a satisfactory level of physical activity, and 57 people (17%) were assessed as performing physical activity a high level.

Table 1 contains dependence between physical activity level regarding sex, age and place of living. In all statistical comparisons *p* < 0.05, but the strongest relation was observed between place of living and physical activity level (V-Cramera = 0.475): 2.5 times more older people living in the institutions had insufficient activity that those living in own houses.

**Table 1:** Level of physical of activity by sex, age and place of living (*N* = 341)

Qualitative variable		Insufficient N(%)	Sufficient N(%)	High N(%)
Sex	Woman	118(34.60)	108(31.67)	47(13.78)
	Man	46(13.49)	12(3.52)	10(2.93)
Pearson test (chi2; df; p; V- Cramera)		**14.38; 2; 0.001; 0.205**		
Age	60–74	91(26.69)	86(25.22)	42(12.32)
	75–89	73(21.41)	34(9.97)	15(4.40)
Pearson test (chi2; df; p; V- Cramera)		**10.56;2;0.005; 0.175**		
Place of living	Own home	46(13.49)	84(24.63)	48(14.08)
	Institution	118(34.60)	36(10.56)	9(2.64)
Pearson test (chi2; df; p; V- Cramera)		**76.98; 2; 0.000; 0.475**		

bold text indicates statistically significant results

### Quality of life

The average quality of life for the whole group of older people as evaluated with the WHOQOL AGE scale was 64.79 (SD = 14.76; min: 18.77-max: 98.07).

Table 2 provides the results concerning the relations between the quality of life and sex age and place of living. Women rated their quality of life higher (65.28) than men (62.41). These results, however, are statistically insignificant. Seniors living in their own homes rated their quality of life higher (69.51) than seniors living in nursing homes (59.59). These results are statistically significant.

Regarding age, people aged 60–74 scored 67.33 points for quality of life. In subscale 1 (satisfaction), the average was 69.21 points, and in subscale 2 (satisfaction of expectations) it was 65.45 points. The people aged 75–89 assessed their quality of life as lower, scoring 60.24. Here, in subscale 1, the average was 61.53 points, and in subscale 2, it was 58.94 points. These results remain statistically significant and are presented in Table 2.

Additionally, the first question (How would you rate your quality of life?) and the third question (How satisfied are you with your health?) from the WHOQOL-AGE questionnaire were analysed in relation to sex, age and place of living. The results revealed a statistically significant correlation between sex, age, place of living and individual quality of life rating, as well as a correlation between age, place of living and health satisfaction rating. Table 2 below provides the results.

**Table 2:** Quality of life (WHOQOL - AGE) by sex, age and place of living (*N* = 341)

Qualitative variable		Quality of life (WHOQOL – AGE)				
		Sum F1 (satisfaction) Mean(SD);min–max	Sum F2 (meeting expectations) Mean(SD);min–max	Total score WHOQOL- AGE Mean(SD);min–max	1st question from WHOQOL – AGE questionnaire	2nd question from WHOQOL – AGE questionnaire
Sex	Woman	67.01(14.06); 17–96	63.55(18.28); 13–100	65.28(14.48); 19–98	3.71(0.77);1–5	3.31(0.93);1–5
	Man	64.25(15.28); 17–88	61.41(19.07); 13–95	62.83(15.83); 26–91	3.52(0.67);2–5	3.08(1.04);1–5
U Mann- Whitney test(U; p)		8649.50; 0.869	8776.00; 0.486	8793.50; 0.502	**7978.00;** **0.048**	8243.00; 0.128
Age	60–74	69.21(12.51); 27–96	65.45(17.69); 17–100	67.33(13.36); 35–98	3.77(0.75);1–5	3.37(0.94);1–5
	75–89	61.53(16.02); 17–92	58.94(19.05); 13–97	60.24(16.06); 19–95	3.50(0.73);2–5	3.08(0.95);1–5
U Mann -Whitney test (U; p)		**9668.00;** **0.000**	**10783.00;** **0.003**	**9988.00;** **0.001**	**10790.00;** **0.001**	**10923.50;0.002**
Place of living	Own home	69.83(12.96); 17–96	69.20(15.57); 19–100	69.51(12.91); 18–98	3.85(0.71);2–5	3.69(0.84);1–5
	Institution	62.74(14.88); 17–90	56.44(19.05); 13–94	59.59(14.96); 26–91	3.47(0.77);0–5	3.33(1.08);0–5
U Mann- Whitney test (U; p)		**10577.00;** **0.001**	**8976.50** **0.000**	**8992.50;** **0.000**	**10716.00;** **0.000**	**12043.50;0.003**

bold text indicates statistically significant results

### The correlation between physical activity and quality of life

Statistical analysis between physical activity and quality of life proved significant dependence for the following: global quality of life rating (*p* = 0.000) and subscales F1 (*p* = 0.001) and F2 (*p* = 0.000). It was concluded that increasing physical activity contributes to QOL improvement. Table 3 below provides the results.

**Table 3:** Correlation between physical activity (IPAQ) and quality of life (WHOQOL - AGE)

Level of physical activity (IPAQ)	Quality of life (WHOQOL - AGE)					
	WHOQOL - AGE; F1 Mean (SD); min–max; Me	Kruskal -Wallis test (H;p)	WHOQOL - AGE; F2 Mean (SD); min–max; Me	Kruskal -Wallis test (H;p)	WHOQOL - AGE Total score Mean (SD); min–max; Me	Kruskal -Wallis test (H;p)
Insufficient	63.16(14.95); 17–94;65.38	**(2,** ***N*** ** = 341) = 17.89; 0.001**	57.52(19.94); 13–94;59.52	**(2,** ***N*** ** = 341) = 23.43; 0.000**	60.34(15.76); 18–94;61.90	**(2,** ***N*** ** = 341) = 25.17; 0.000**
Sufficient	68.36(13.52); 29–96;71.15		67.50(15.68); 26–97;67.85		67.93(12.97); 17–94;69.27	
High	71.86(11.81); 36–96;73.07		69.90(14.32); 34–100;67.85		70.88(11.26); 52–98;68.95	

bold text indicates statistically significant results

Additionally, the Spearman rank correlation coefficient test was applied to investigate the occurrence of a correlation between quality of life and physical activity (Table 4). The results indicate a weak positive correlation between total physical activity and three of its components (moderate physical activity, vigorous physical activity and walking). No correlation (*p* > 0.05) between time spent sitting and quality of life components or total quality of life results was observed.

**Table 4:** Correlation between quality of life (WHOQOL - AGE) and physical activity level (IPAQ) measured with Spearman’s rcc

Components of physical activity (IPAQ)	Quality of life (WHOQOL – AGE)		
	WHOQOL – AGE Score F1	WHOQOL – AGE Score F2	WHOQOL – AGE Total score
Physical activity total average (SD) MET(metabolic equivalent) -min/week	**< 0.05, 0.270**	**< 0.05, 0.275**	**< 0.05; 0.302**
Moderate physical activity average (SD) MET(metabolic equivalent) -min/week	**< 0.05; 0.215**	**< 0.05; 0.173**	**< 0.05; 0.211**
Vigorous physical activity average (SD) MET(metabolic equivalent) -min/week	**< 0.05; 0.189**	**< 0.05; 0.136**	**< 0.05; 0.175**
Walking average (SD) MET(metabolic equivalent) -min/week	**< 0.05; 0.253**	**< 0.05; 0.258**	**< 0.05; 0.283**
Time spent sitting average (SD) MET(metabolic equivalent) -min/week	> 0.05; no correlation	> 0.05; no correlation	> 0.05; no correlation

bold text indicates statistically significant results

Table 5 presents results about physical activity and it components in related to places of living- own house flat and nursing homes. Statistically significant correlations occurred in all elements of physical activity. Living in your own home definitely results in higher physical activity results. The only exception is sedentary behaviour, place of residence does not matter.

**Table 5:** Physical activity in related to place of residence

Components of physical activity (IPAQ)	Place of residence		
	Own house/flat Mean (SD) (min-max)	Nursing homes Mean (SD) (Min-max)	U Mann- Whitney test (U; p)
Physical activity total	2105.62 (2339.06) (0.00-11358.00)	591.50 (1017.11) (0.00-5439.00)	**6405.00; <0.001**
Moderate physical activity	617.03 (1093) (0.00-7200.00)	175.95(548.49) (0.00-5040.00)	**9471.00; <0.001**
Vigorous physical activity	526.51(1339.80) (0.00-10400.00)	52.76 (290.03) (0.00-2400.00)	**10607.50; <0.001**
Walking	1043.56 (1136.68) (0.00-6930.00)	398.92(834.73) (0.00-6390.00)	**7499.00; <0.001**
Time spent sitting	215.69 (115.07) (30.00-600.00)	195.57(123.96) (10.00-600.00)	3815; >0.05

bold text indicates statistically significant results

## Discussion

Total physical activity was assessed by means of the IPAQ international questionnaire and amounts to 1381.87 ± 1978.60 MET-min/week. Slightly lower results of the study amounting to 1372.4 ± 1396.6 kcal based on the IPAQ questionnaire were obtained by Kim Y.J. et al. in a group of Korean older people [14]. Żurek et al. applied the IPAQ questionnaire in his study and showed that 50% of older women performed a sufficient amount of physical activity, whereas less than 25% of the women questioned reported performing vigorous physical activity [15]. Moreover, Puciato D. et al., in his research based on the IPAQ and conducted among respondents aged 59.1 ± 2.9, obtained a higher proportion of respondents declaring a high level of physical activity (42.3%), followed by 36.2% of participants stating a moderate level of physical activity and 21.4% with a low level of physical activity [16]. Some discrepancies between the results of the study described herein and the results scored by Puciato D. et al. arise from the younger age of the surveyed group. In a Scottish study by Tomaz et al., 43% of older people had a high level of physical activity, 36% moderate and 21% low [17]. On the other hand, in the study by Larsen et al. conducted in Denmark, older people had lower physical activity: 27.3% of them were at a high level of physical activity, 33.3% at a moderate level and 39.4% at a low level [18].

The study discussed herein proved that the QOL of people aged 60 and over assessed with the WHOQOL-AGE questionnaire amounts to 64.79. Jurkiewicz et al., however, claimed higher results for quality of life among physically active older women. In addition, post hoc analysis showed that physically active respondents demonstrated significantly higher quality of life than those performing irregular physical activity [19]. Fidecki W. et al. evaluated quality of life in a group of 264 people aged 65–93. Their results assessed with the WHOQOL-AGE scale were at the level of 74.14 ± 15.31, whereas in subscale 1, they obtained a higher score of 71.11 ± 13.88 than in subscale 2–69.15 ± 18.55 [20]. Moreover, Bartoszek A. et al. examined quality of life using the WHOQOL-AGE scale and scored the average quality of life result of 70.14 ± 15.31, in subscale 1–71.11 ± 13.88, and subscale 2–69.15 ± 18.55 [21]. Kowalczyk et al. examined the quality of life with the WHOQOL-AGE scale in a group of 1008 older people and obtained the average result of 67.20, and their scores in subscale 1 were also higher – 70.78, and in subscale 2–69.09. Social and demographic factors such as age (*p* < 0.000), education (*p* < 0.000), place of living (*p* < 0.029), marital status (*p* < 0.000), financial condition (*p* < 0.000), status of living (*p* < 0.019) and physical condition (*p* < 0.000) determined the quality of life of this group in a statistically significant way [22]. In our research, however, age was the main differentiator for the quality of life score – younger older people declared higher quality of life assessed both by means of the total score and a subjective result (question no. 1 in WOQOL- AGE questionnaire).

Importantly, our research revealed a tendency for the lower results in subscale 2, which refers to meeting expectations, particularly those concerning social relationships. Our research study proved that quality of life in subscale 2 is lower than life satisfaction in subscale 1. Older people seem to be more willing to accept deteriorating physical conditions, lowering efficiency or physical capacity rather than the loss of social functions, social relationship worsening or lower mental well-being.

Obviously, in addition to the WOQOL-AGE test, there are some other questionnaires available to evaluate the quality of life for older people and the impact of physical activity on older people’s quality of life. The cross-sectional study conducted by Puciato et al. on a group of 1000 participants proved that global quality of life, health state, and the quality of life concerning physical, psychological, social and background aspects were by far better in the group of physically active participants [16]. The study conducted by Niedermeier et al. on the correlation between physical activity and quality of life with the use of the WOQOL-BREF confirmed a positive correlation between physical activity and all spheres of quality of life [23]. Additionally, the authors of the study emphasized the importance of social relations in the context of the quality of life of older people. Daimiel et al.’s research, known as the PREDIMED – plus trial, performed with the SF36 questionnaire, indicated that both a higher level of physical activity and physical capacity are firmly connected with a better level of quality of life (higher results were obtained in all domains of the SF36 questionnaire) [24]. Similarly, Oh et al. examined the impact of the three most representative activities (including resistance, resilience and walking) on quality of life in an older dwelling group in a particular community. They noticed that quality of life parameters, including mobility, self-care, routine activities, pain/discomfort and fear/depression assessed with EuroQOL, improved [25]. Furthermore, Uniatowska and Kupczyk examined factors differentiating the level of functional efficiency for older people (*n* = 509) evaluated by means of FFFT and confirmed a higher level of functional efficiency among physically active individuals (both men and women) [26].

Lepsy et al.’s study indicated the importance of maintaining good physical condition and the impact it has on quality of life, especially in the group of people aged 80 and above [27]. Subsequently, Esain et al. pointed out the consequences of giving up physical activity for this group. It is a period of a three-month break, which may lead to a decrease in dynamic balance and quality of life [28]. This study also confirms the effect of physical activity on maintaining good health and well-being, which can lead to quality of life improvement. It is worth mentioning that the two research tools, that have been applied to this study had never been used together. The IPAQ questionnaire is commonly implemented to reveal the effect of physical activity on a particular somatic disease or mental health. Interestingly, it has rarely been used to investigate the impact of physical activity on quality of life as assessed with the WHOQOL-AGE questionnaire. The issue analysed herein is essential in the light of an aging society and requires further investigation, particularly in the oldest group.

There was noticed, that low level of physical activity and a high proportion of the day sedentary lifestyle are reported by older people living in nursing homes. Keogh et al. indicated that even 12.9 h average during the day older residents spent sitting [29]. The reasons for these results are unclear. On the one hand, people in nursing homes are more sick, inactive, have low of self-efficacy and perceived severity [30], that are related to the level of physical activity. On the other hand, they have wider access to professional rehabilitation and sports activities conducted by trainers. These conditions should reduce the differences between physical activity among nursing homes residents and older people living in own homes, but our research does not indicate.

The strength of the study is the comparison of two questionnaires regarding quality of life and physical activity (in which the WHOQOL-AGE questionnaire is dedicated only to older people), which has not yet appeared in the available literature. Another strength of the study is the comparison of two completely different groups, such as nursing homes and own homes/flats. Theoretically, nursing homes residents have 24/7 medical care, access to rehabilitation, a doctor, meals are prepared in accordance with nutritional recommendations, access to organized activities dedicated to them, and yet their quality of life is lower than that of people who only participate in U3A classes.

However, during the research, were noted several limitations. First, older people often had problems completing the questionnaire; usually, these problems concerned the correct quantitative values describing performed activities. As a consequence, the number of correctly completed questionnaires decreased by almost 50% (600 were distributed and 341 were completed in total). Second, a questionnaire for people aged 60 and above that takes into account their activity is needed because the short IPAQ version does not contain age restrictions. Third, it is worth noting that an increasing number of older people, despite retirement, still work professionally, and this variable should be included in the description of their physical activity. In addition to constructing tools assessing the activity of older people, attention should be given to a more precise distinction between sitting and resting after physical activity.

## Conclusion

1. Physical activity improves quality of life among people aged 60–89. It is worrying, however, that every third older person questioned declares no physical activity or walking, whereas every second respondent obtains an insufficient amount of physical activity.
2. Age significantly affects quality of life for older people. Older people aged 74 and below enjoy a higher quality of life than people aged 75 and above. Similarly, individual quality of life scores were higher in the group of younger older people. Sex had no significant effect on quality of life, as evaluated with the WHOQOL-AGE, although the subjective quality of life assessment of females was higher than that of males.
3. The people aged 60 and over scored higher on subscale F1 (satisfaction) than on subscale F2 (meeting expectations) in both surveyed age groups.
4. Older people have an insufficient level of physical activity, most of whom are men, people 75 + and residents of institutions.
5. Physical activity people 60 and above is strongly related to place of living. Total physical activity and it components are even 5 times higher among people who are living in their own homes/flats. Only time spent sitting is no statistically significant. People, who are living in nursing homes reported low levels of physical activity, despite to availability to physical exercises and professional rehabilitation and more opportunities to take part in organized activities.

Practical application of research results:

1. The results obtained can be used to create educational programs and campaigns that increase people over 60’ readiness to undertake appropriate behaviors related to physical activity, which improve healthy lifestyle, quality of life and personal independence in everyday activities.
2. A still current challenge for medical staff and caregivers of older people is to activate them to physical activity, because regardless of where they live, they achieve high indicators measuring a “sedentary lifestyle”.

## Data Availability

The datasets used and/or analysed during the current study are available from the corresponding author upon reasonable request. Corresponding author: Aleksandra Kiełtyka-Słowik; email: aleksandra.kieltyka@awf.krakow.pl.

## Abbreviations

ACC: American College of Cardiology
AHA: American Heart Association
CTT: classical test theory
IADL: Instrumental Activities of Daily Living Scale
IPAQ: International Physical Activity Questionnaire
IRT: item response theory
MET: minutes a week
PA: physical activity
QOL: quality of life
U3A: University of Third Age
WHOQOL-AGE: World Health Organization Quality of Life—Age
